# Chemical Recycling of Polyethylene Terephthalate (PET) Medical Waste for the Sustainable Production of Biomedical Materials

**DOI:** 10.3390/jfb17070339

**Published:** 2026-07-13

**Authors:** Haoming Yang, Yuan Yu

**Affiliations:** School of Safety Science and Engineering, Nanjing Tech University, Nanjing 211816, China; yanghaoming@njtech.edu.cn

**Keywords:** polyethylene terephthalate (PET), chemical recycling, catalytic pyrolysis, enzymatic recovery, biomedical materials

## Abstract

This study systematically evaluates the application prospects of three chemical recycling technologies for resource recovery from PET medical waste and the sustainable production of biomedical materials: catalytic pyrolysis, thermochemical recovery, and enzymatic hydrolysis. Orthogonal experimental designs and Box–Behnken response surface methodologies were used to optimise process parameters, and an extended assessment platform covering chemical purity, molecular weight distribution, biocompatibility, and mechanical properties was established. Under optimised conditions (200 °C, 3% *w*/*w* catalyst, 4 h, 6:1 ethylene-glycol-to-PET mass ratio), catalytic pyrolysis with zinc acetate achieved a terephthalic acid (TPA) recovery of 92.3 ± 1.8% at a product purity of 98.2 ± 0.5%, and retained 97.6% of the tensile strength and 97.4% of the elastic modulus of virgin PET. Although the enzymatic process was relatively long at 24 h, it had the best biocompatibility (L929 fibroblast viability 94.1 ± 2.2% and haemolysis 1.82 ± 0.28%) and reduced the carbon footprint by 46.5% compared to catalytic processing. Thermochemical recovery was completed in 1 h at 500 °C, achieving a TPA recovery of 71.2 ± 3.8%, and is suitable for large-scale processing of low-value medical waste streams. Biocompatibility tests showed that PET regenerated via the three paths met the ISO 10993 series of standards, with a cytotoxicity grade of 0–1 and an endotoxin content below 0.5 EU/mL. Gel permeation chromatography showed that the number-average molecular weight (M_n_) of chemically recycled PET was between 21,200 and 24,100 g·mol^−1^ (compared to 24,500 g·mol^−1^ for virgin PET), approximately 86.5% to 98.4% of the virgin value, and significantly higher than mechanically recycled PET. The technical route and quality-control system established here provide a scientific basis for the closed-loop recycling of medical-grade PET and support the green transformation of the medical industry.

## 1. Introduction

Polyethylene terephthalate (PET) is one of the highest-production thermoplastics in the world, with an annual production exceeding 70 million tonnes [1]. Given its excellent biocompatibility, mechanical strength, and chemical stability, it is suitable for the construction of artificial vascular conduits, cardiac-valve reinforcement rings, surgical meshes, and many other systems used in biomedicine [2]. By 2022, the world’s PET market had reached 25.47 million tonnes and is expected to grow to about 35.7 million tonnes by 2030 through increasingly diverse applications [3].

Despite these advantages, PET is now an environmental problem. About 460 million tonnes of plastic waste are produced every year worldwide, and only about 9% have been reused. Of the remainder, 50% are sent to landfills, 19% are burned, and the rest enter the natural environment [4]. In 2024, global plastic waste production reached 220 million tonnes, an increase of 7% relative to 2021.

Chemical recycling can be used to reduce pollution from waste. Mechanical recycling is restricted to a limited number of cycles before serious property degradation, whereas chemical recycling can fully depolymerise PET into its monomer components for closed-loop material recycling [5]. It also operates in a relatively mild environment, has a lower energy requirement, and does not need harmful chemicals or expensive equipment [6]. For biomedical applications, chemical recycling can produce high-purity monomers suitable for the production of medical-grade PET that meets strict safety and performance requirements [7,8].

A full-fledged chemical recycling system is put forward in this paper to address the technological deficiencies of recycling medical-grade PET waste. Three major technologies are explored: (i) catalytic pyrolysis to achieve a monomer recovery rate above 90%; (ii) thermochemical recycling via optimised pyrolysis for the production of reusable chemical feedstocks; and (iii) enzymatic hydrolysis using room-temperature alkaline pretreatment followed by enzymatic degradation for high-efficiency recycling at low energy and environmental cost [9,10]. Process optimisation covers a reaction temperature of 30–700 °C and a system pressure of 1–40 bar, as well as the selection of catalysts and purification by crystallisation, distillation, and membrane separation.

Several problems remain to be solved. High-purity requirements for medical-grade PET recycling include a monomer purity above 97%, strict compliance with ISO 10993-5 cytotoxicity standards, haemolysis below 5%, and the United States Pharmacopeia’s maximum heavy-metal limits [11]. Each recycling technology has its own drawbacks: catalytic pyrolysis needs accurate reaction control, thermochemical methods are energy intensive, and enzymatic approaches face challenges in reaction rate and cost. In light of these problems, the present study builds an all-encompassing performance-evaluation system for chemical purity, molecular weight distribution, biocompatibility, and mechanical properties, thereby providing scientific support for quality control in the production of medical-grade recycled PET.

## 2. Materials and Methods

### 2.1. Materials

Three different sources of PET waste were collected to comprehensively assess the feasibility of chemical recycling technology for various biomedical PET materials. Medical waste PET was taken from three tertiary hospitals and included discarded vascular grafts, infusion-device housings, and various syringe packaging materials [12]. The material was sterilised in an autoclave at 121 °C for 30 min to remove any living organisms. FTIR was used to identify the chemical structure, and differential scanning calorimetry (DSC) was employed to determine thermal-behaviour changes. Sample purity was >95%.

Industrial-grade PET was obtained from two medical-device companies in the form of injection-moulding scraps, quality-control rejects, and production cutting residues, with an initial purity of >98%. It was then mechanically crushed into uniform particles (2–5 mm). The third category was mixed waste PET containing additives such as antioxidants, UV stabilisers, and plasticisers, with mass fractions of 0.5–2.0% according to thermogravimetric analysis (TGA) and gas chromatography–mass spectrometry (GC–MS).

Zinc oxide (99.9%), zinc acetate (analytical grade), 1-butyl-3-methylimidazolium chloride ([BMIM]Cl, >99%), and *p*-toluenesulfonic acid (99%) were employed as catalysts. PETase (specific activity >100 U/mg) and MHETase (specific activity >80 U/mg) were heterologously expressed in an *Escherichia coli* system from *Ideonella sakaiensis* [13,14]. PBS and Tris-HCl buffers with different pH values were used as buffers for enzyme-activity experiments.

### 2.2. Catalytic Pyrolysis Optimisation

The four main factors identified by single-factor experiments were reaction temperature, catalyst dosage, reaction time, and alcoholysis-agent-to-PET mass ratio. An L9(3^4^) orthogonal experimental design was then used to systematically study the impact of reaction temperature (180–220 °C), catalyst dosage (1–5% of PET mass), reaction time (2–6 h), and ethylene-glycol-to-PET mass ratio (4:1 to 8:1) on monomer recovery. Each test was conducted in triplicate [15].

Box–Behnken response surface methodology was used to optimise the two principal factors (reaction temperature and catalyst dosage). A second-order polynomial regression model incorporated linear, quadratic, and interaction terms. The reliability of the model was verified through ANOVA and residual analysis, and R^2^ > 0.95 was required for the prediction and experiment. A 250 mL three-necked flask was used as the reaction vessel, nitrogen was added to provide an inert atmosphere, and mechanical stirring was carried out at 300 rpm; TPA was then recovered by acidification, recrystallisation, and vacuum drying.

### 2.3. Thermochemical Recovery of PET

Five temperature points in the range of 300–700 °C were set for the experiment, and the impact of different heating rates (5, 10, and 20 °C/min) on product distribution was examined. A high-precision temperature-controlled custom tubular-furnace reactor and a three-stage condensation collection system were constructed. Nitrogen gas at 50–200 mL/min was used to prevent oxidation. Each experiment used 10 ± 0.1 g of PET. Three condensers were set at 25 °C, 0 °C, and −20 °C to collect fractions with different boiling points. Vacuum distillation was used to further purify the condensed products, which were then characterised by GC–MS, FTIR, and ^1^H-NMR.

### 2.4. Enzymatic Recycling Process

Enzymatic recycling was performed in two steps. The first stage was alkaline pretreatment, in which the sodium-hydroxide concentration was set to 0.1–1.0 M, and parameters such as temperature (room temperature to 60 °C) and time (0.5–2 h) were varied systematically. Environmental scanning electron microscopy (ESEM) and surface profiling by atomic force microscopy (AFM) were carried out, and BET nitrogen-adsorption analysis was also performed. The second stage examined the cooperative effect of PETase and MHETase by adjusting their mass ratio (1:1, 2:1, and 1:2), reaction pH (7.0–9.0), temperature (30–40 °C), and total enzyme loading (50–200 U/g PET). A 50 mL conical flask was shaken at 150 rpm, and TPA accumulation was measured at various times by high-performance liquid chromatography (HPLC).

### 2.5. Scale-Up Experiments

Three progressively larger-scale experiments were carried out to verify the laboratory results. A 100 mL three-necked flask with a magnetic stirrer was used for the laboratory-scale experiment; a 1 L glass reactor with adjustable mechanical stirring (200–500 rpm) was used for the small-scale experiment; and a 10 L stainless-steel reactor with precise temperature control and multiple online monitors was used for the pilot-scale experiment. A stability test was run for 72 h with samples taken at 8 h intervals. The process was stable and met the requirements: the coefficient of variation of monomer recovery was below 5% and the standard deviation of product purity was below 2%. Energy consumption per unit of recovered PET and operating-temperature stability were also recorded.

### 2.6. Characterisation

HPLC (Waters 2695, C18 column, methanol/water mobile phase containing 0.1% formic acid, detection at 254 nm) and GC–MS (Agilent 7890B-5977A, DB-5MS column) were used to determine the quantitative chemical composition. A Bruker AVANCE III (600 MHz) instrument was employed to carry out ^1^H-NMR and ^13^C-NMR experiments for product-structure verification. Trace impurities were identified by liquid chromatography–tandem mass spectrometry (LC–MS/MS), and heavy-metal contents were measured by inductively coupled plasma–mass spectrometry (ICP–MS). LC–MS/MS (residual organic by-products) and ICP–MS (Zn, Sb, Pb, Cd, and other heavy metals), performed on both the crude and the single-recrystallised product to quantify purification efficiency; the corresponding concentrations before and after purification, instrument detection limits, and compliance with the United States Pharmacopeia heavy-metal limits are reported in the paired purity table. Molecular-weight parameters were obtained by gel permeation chromatography (GPC, Waters 1515 with a refractive-index detector) using tetrahydrofuran as the elution solvent [16].

Following ISO 527, mechanical properties were measured at 5 mm/min on a universal testing machine under controlled conditions (23 ± 2 °C and 50 ± 5% RH), with a minimum of five parallel specimens per material. Accelerated ageing was performed at 70 °C and 75% relative humidity for 30 days (approximately 5 years at 37 °C).

#### 2.6.1. Cytotoxicity and Biocompatibility

According to ISO 10993-5, L929 mouse fibroblasts (obtained from the Cell Bank of the Chinese Academy of Sciences, Shanghai, China; Cat. No. TCM38) were used in MTT cytotoxicity assays. Material extracts (100%, 75%, 50%, and 25%) were prepared at an extraction ratio of 0.2 g/mL in DMEM at 37 °C for 24 h and incubated with cells for 48 h [17]. According to ISO 10993-4, fresh rabbit blood was diluted 1:1.25 in PBS and contacted with material extracts at 37 °C for 1 h, and haemoglobin release was determined at 545 nm. Bacterial-endotoxin concentration was determined by dynamic turbidimetry (target < 0.5 EU/mL). Subcutaneous implantation in Sprague–Dawley rats was used to examine the in vivo tissue response over 4 weeks, and histology of the fibrous capsule around the implant was conducted.

#### 2.6.2. Antibacterial Activity and Degradation

*Staphylococcus aureus* (Gram-positive) and *Escherichia coli* (Gram-negative) were selected for a colony-forming-unit (CFU) counting assay. Bacterial suspensions (10^6^ CFU/mL) were added to the surface of the material, incubated at 37 °C for 24 h, and the colonies were counted. Degradation experiments were carried out in PBS buffer (pH 7.4) at 37 °C for 12 weeks; at 2-week intervals, mass loss, molecular-weight retention, and pH changes were recorded. SEM and FTIR were used to observe surface morphology and degradation products [18].

### 2.7. Statistical Analysis

SPSS 26.0 was used for the statistical analysis. Data are presented as mean ± standard deviation (n≥3). Specifically, n=6 independent replicates were used for the MTT cytotoxicity and haemolysis assays, n=3 for endotoxin tests, n=5 specimens for mechanical tests, and n=6 animals per group for subcutaneous implantation; each catalytic-pyrolysis condition was performed in triplicate. A post hoc power analysis (G*Power 3.1, α=0.05) determined that these sample sizes had statistical power >0.80 for the main inter-route comparisons of TPA recovery, cell viability, and tensile strength. One-way ANOVA and Duncan’s multiple-comparison test were used to determine inter-group differences, and a *p*-value below 0.05 was considered statistically significant. R^2^ values, adjusted R^2^ values, predicted R^2^ values, and lack-of-fit tests of the response-surface fits were assessed. Life-cycle assessment (LCA) followed ISO 14040 standards using SimaPro 9.0 software with the Ecoinvent 3.6 database [19]. Net present value (NPV) and internal rate of return (IRR) were employed to assess economic feasibility, and a sensitivity analysis was carried out on major risk factors. The LCA used a cradle-to-gate system boundary and a functional unit of 1 kg of medical-grade recycled PET resin; the inventory included feedstock collection and sterilisation, reagent and catalyst inputs, heterologous enzyme production (with a carbon footprint allocated on a mass basis to the recovered monomers), reaction energy, purification, and on-site emissions, with virgin fossil-based PET as the comparison baseline. One-at-a-time sensitivity analysis was carried out for enzyme dosage, enzyme-production effect, electricity mix, and monomer yield, and the reported 46.5% carbon-footprint reduction of the enzymatic route corresponds to the optimised-yield scenario compared with catalytic processing at this boundary [20,21].

## 3. Results

### 3.1. Catalytic Pyrolysis Performance

**Figure 1:**
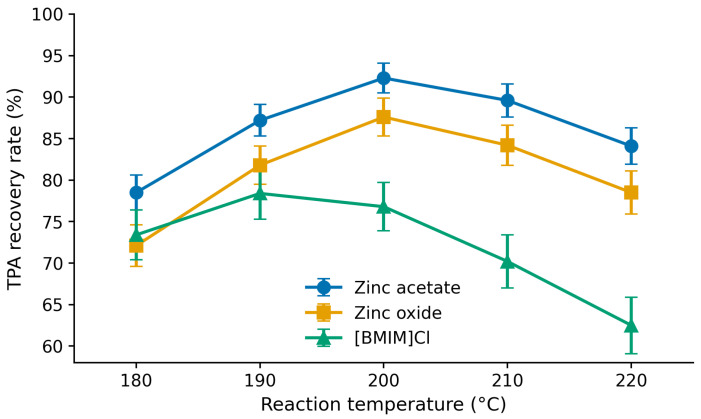
Effect of reaction temperature on TPA recovery rate for three catalysts (zinc acetate, zinc oxide, and [BMIM]Cl) at 3% *w*/*w* catalyst dosage and 4 h reaction time. The optimum is 200 °C with zinc acetate (92.3 ± 1.8%).

A single-factor screen determined that the two main reasons for TPA-recovery fluctuations were changes in reaction temperature and catalyst addition. As shown in Figure 1, the three catalysts produced three different temperature curves. Zinc acetate achieved a TPA recovery of 92.3 ± 1.8% at 200 °C, the highest among the systems tested; zinc oxide reached 87.6 ± 2.3% under the same conditions, likely due to its poor solubility and dispersion in the reaction system [22]. The ionic liquid [BMIM]Cl was active at a lower temperature (peaking at 190 °C), but TPA recovery was still limited to 78.4 ± 3.1%, so further activation or modification is required for it to be competitive.

Box–Behnken response-surface analysis revealed a clear optimum. The fitted second-order polynomial had an R^2^ of 0.978 and an adjusted R^2^ of 0.961, with no significant lack of fit (p=0.18). The optimum was at 200 °C and 3% (*w*/*w*) catalyst addition, exactly matching the predictions of single-factor analysis, and a TPA recovery of 92.6% was obtained experimentally (92.3 ± 1.8%). Under these optimised conditions (3% *w*/*w* zinc acetate, a reaction time of 4 h, and a 6:1 ethylene-glycol-to-PET mass ratio), the purity of the product after one recrystallisation was 98.2 ± 0.5%. Co-recovered ethylene glycol (EG) was obtained in a yield of 89.7 ± 2.1% and a purity of 97.6 ± 0.7%, and both monomers were used for subsequent polycondensation.

PET source affected recovery efficiency. Autoclaved PET medical waste showed a decrease in crystallinity from 32.4% to 28.7% and an increase in the amorphous fraction, which promoted depolymerisation; under the same conditions, the monomer recovery was 5–8% higher than that for industrial scrap. PET with additives showed lower recovery due to interactions between the additives and the catalyst, which reduced catalytic activity; increasing the catalyst dosage to 5% or extending the reaction time to 6 h compensated for this to some extent [23].

### 3.2. Thermochemical Recovery Yields

**Figure 2:**
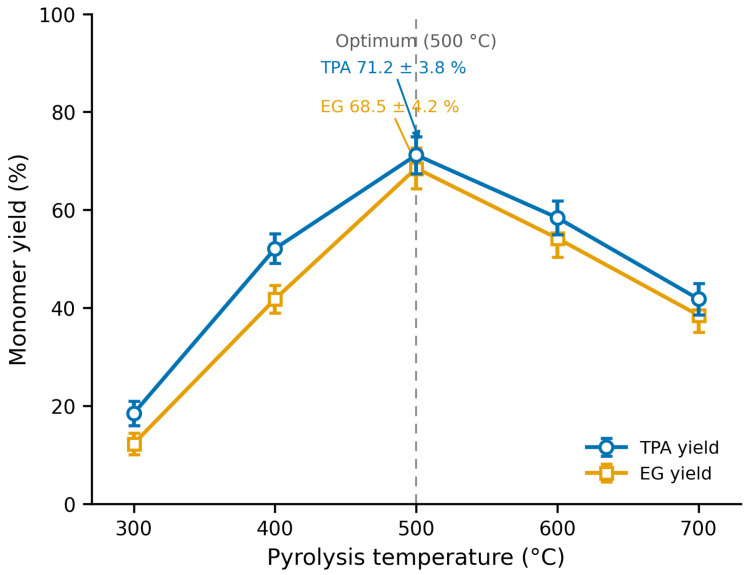
Yields of TPA and EG monomers as a function of pyrolysis temperature (300–700 °C, 10 °C/min, N_2_ atmosphere). The highest yields occur at 500 °C, with TPA and EG yields of 71.2 ± 3.8% and 68.5 ± 4.2%, respectively.

Pyrolysis temperature affected the distribution of products (Figure 2). At 300 °C, conversion was incomplete, so the product mixture contained a considerable amount of oligomeric BHET and unreacted PET. From 400 °C, depolymerisation increased rapidly, and the yield of TPA was 52.1% and that of EG was 41.8%. The optimum was 500 °C, with the highest balance of monomer yields (TPA 71.2 ± 3.8% and EG 68.5 ± 4.2%) and only a small amount of secondary products. Above 550 °C, secondary cracking accelerated, so TPA was rapidly lost as gaseous products and char. At 700 °C, the yield of TPA dropped to 41.8% and the ratio of gas/char to feed mass exceeded 41%.

Heating rate also affected product yield. At 5 °C/min, the yield of TPA reached 74.5 ± 2.6%, slightly higher than that obtained at 10 °C/min, because the longer residence time in the optimal range promoted monomer generation. Increasing the rate to 20 °C/min reduced both the TPA and EG yields (to 63.8 ± 3.5% and 60.2 ± 4.0%, respectively) due to an accelerated onset of secondary cracking. GC–MS analysis of the 500 °C product showed by-products such as benzoic acid (3.2%) and acetaldehyde (2.1%), which were removed by vacuum distillation to obtain a final TPA purity of 92.4 ± 1.1%. Although the product range for catalytic pyrolysis is wider, the short reaction time (1 h) and the absence of a catalyst still make thermochemical recovery economically feasible for high-volume, low-value-added medical waste streams.

### 3.3. Enzymatic Recycling Efficiency

Alkaline pretreatment significantly increased enzyme accessibility. Treatment with 0.5 M NaOH for 2 h at room temperature increased the surface roughness Ra from 0.32 ± 0.05 μm to 1.87 ± 0.12 μm (a 5.8-fold increase) and increased the BET specific surface area from 0.24 m^2^/g to 2.16 m^2^/g (a 9.0-fold increase). SEM micrographs show uniform nano-pits on the surface, and AFM data indicate no significant fractures in the topography; thus, the material maintained its initial mechanical properties after treatment.

**Figure 3:**
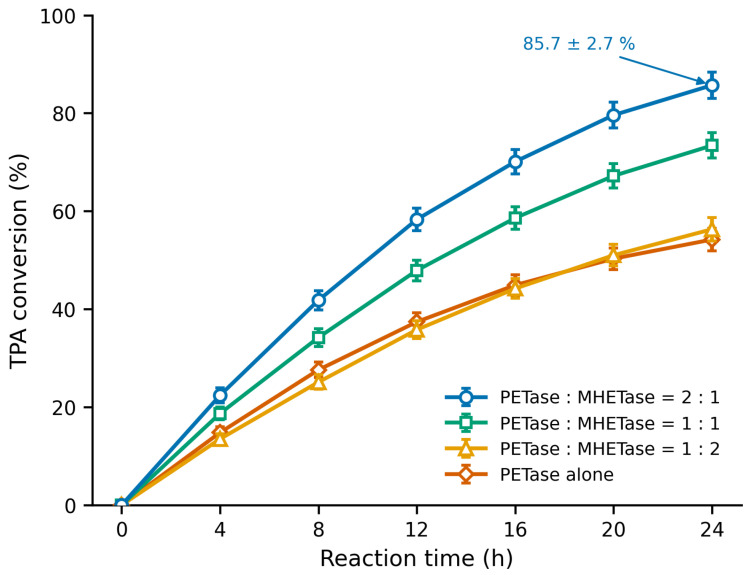
Time course of TPA conversion at different PETase:MHETase mass ratios (total enzyme dosage 150 U/g PET, pH 8.0, 35 °C, after alkaline pretreatment). A 2:1 PETase:MHETase ratio achieved the highest 24 h conversion rate of 85.7 ± 2.7%.

The synergistic action of PETase and MHETase significantly increased the conversion rate (Figure 3). At a 2:1 PETase:MHETase mass ratio, a total enzyme dosage of 150 U/g PET, pH 8.0, and 35 °C, TPA conversion reached 85.7 ± 2.7% in 24 h. Reducing the MHETase loading (1:2 ratio) resulted in the accumulation of the intermediate MHET and a ∼35% decrease in the apparent reaction rate. PETase alone only reached a plateau of 54.2% after 24 h, so MHETase is required to increase the reaction rate over the rate-limiting MHET intermediate (consistent with the two-enzyme mechanism established in Refs. [13,14]). The product purity was 97.8 ± 0.6%, with no organic impurities larger than 500 Da detectable by LC–MS/MS. Critically, the enzymatic reaction occurs at a low temperature of 35 °C and reduces the energy consumption of catalytic pyrolysis by 65%, making it highly energy-efficient for the production of biomedical-grade materials that require minimal pollution. The optimised conditions and monomer-recovery efficiencies of the three chemical recycling routes are summarised in Table 1.

### 3.4. Scale-Up Validation

Scale-up processing confirmed the stability of the catalytic-pyrolysis route at the 10 L pilot-scale reactor level (Table 2). The TPA recovery rate dropped slightly with scale, from 92.3 ± 1.8% at the laboratory level to 88.7 ± 2.4% at the pilot stage, but the product purity remained above 96.8%. Process consistency, as shown by the coefficient of variation (CV) of monomer recovery in nine replicate runs, rose from 1.95% to 2.71% but stayed well below the 5% target, confirming reproducibility at the engineering scale. The energy consumption per kilogram of recovered PET decreased from 1.85 kWh at the laboratory scale to 1.48 kWh at the pilot scale, a reduction of 20% due to a smaller surface-to-volume ratio and less heat loss in the larger system. The continuous 72 h stability test showed no significant drift in operating temperature (±2.5 °C) or recovery rate, qualifying the process for an industrial-scale closed-loop recycling line.

### 3.5. Characterisation of Recycled PET

Collectively, the characterisation studies covered molecular weight and mechanical properties, as well as the biological response and degradation behaviour of recycled PET samples. As shown in Table 3, all three chemical routes produced PET regenerated with molecular weights close to those of virgin PET (by GPC). Catalytic-pyrolysis-recycled PET achieved an M_n_ of 23,800 ± 610 g·mol^−1^, an M_w_ of 47,500 ± 920 g·mol^−1^, a polydispersity index (PDI) of 2.00 ± 0.05, and an intrinsic viscosity [η] of 0.80 ± 0.03 dL/g, meeting the injection-moulding-grade requirement of [η] > 0.75 dL/g. Enzymatic-recycled PET achieved the narrowest distribution (PDI 1.98 ± 0.03) and an M_n_ of 24,100 ± 480 g·mol^−1^, only 1.6% lower than virgin PET; thermochemical-recycled PET had a slightly broader distribution (M_n_ 21,200 ± 980 g·mol^−1^; PDI 2.09 ± 0.08), reflecting the secondary cracking observed in Section 3.2. By contrast, reference data for mechanically recycled PET had a much lower M_n_ (18,600 ± 1420 g·mol^−1^) and a wider PDI (2.12 ± 0.11), indicating that chemical recycling is structurally superior (consistent with GPC data for mechanically reprocessed grades reported in Refs. [16,23]).

Mechanical properties followed the molecular-weight trends. Catalytic-pyrolysis-recycled PET retained 97.6% of the tensile strength and 97.4% of the elastic modulus of virgin PET (Table 3). Enzymatic-recycled PET had a tensile strength of 67.8 ± 2.1 MPa and an elongation at break of 122 ± 7%, exhibiting good strength–toughness properties for medical fibres and films. Thermochemical-recycled PET had a tensile strength of 62.3 ± 3.1 MPa and a modulus of 2.25 ± 0.11 GPa, still exceeding the mechanical performance targets adopted here for vascular-graft fibres (tensile strength > 50 MPa, modulus > 2.0 GPa), consistent with the evaluation framework for tubular vascular prostheses defined in ISO 7198. Fatigue tests at 50% of the ultimate tensile strength showed that catalytic and enzymatic samples reached a life exceeding $1\times 10^{6}$ cycles without failure, making them suitable for cardiovascular implants. Accelerated ageing (70 °C, 75% RH, 30 d, equivalent to 5 years at 37 °C) reduced the tensile strength of catalytic-pyrolysis-recycled PET by only 5.8% and the molecular weight by 7.2%, indicating long-term stability in service.

**Figure 4:**
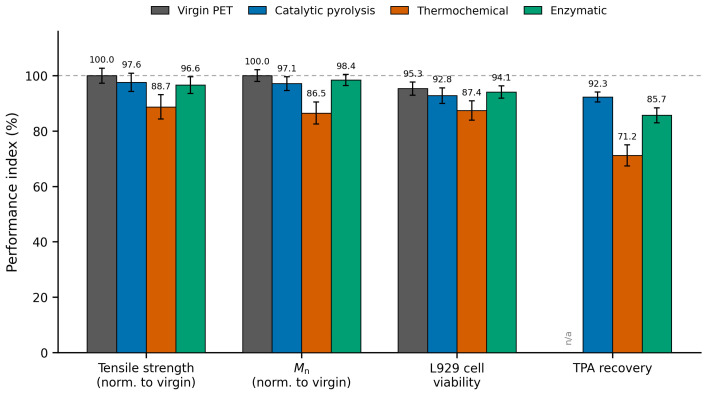
Comparison of virgin and recycled PET based on four key performance indicators. Tensile strength and M_n_ are normalised to virgin PET; cell viability and TPA recovery are in absolute percentages. Error bars are standard deviations.

Figure 4 shows the four major performance indices of recycled PET versus virgin PET. Catalytic-pyrolysis and enzymatic recycling of PET are within 3–8% of virgin PET for tensile strength, molecular weight, cell viability, and TPA recovery, whereas thermochemical recycling of PET is about 10–13% away on structural and biological indicators. The monomer-recovery rates of catalytic pyrolysis (92.3%), enzymatic recovery (85.7%), and thermochemical pyrolysis (71.2%) are the highest among the three routes, and all are relatively mild processes yielding high-purity products.

#### 3.5.1. Cytotoxicity and Biocompatibility Results

Biocompatibility was assessed by MTT cytotoxicity, haemolysis, endotoxin tests, and subcutaneous implantation (Table 4). All recycled PET samples met the ISO 10993-5 threshold of L929 cell viability ≥70% for all extract concentrations. Enzymatic-recycled PET had the highest cell viability (94.1 ± 2.2%), which was not statistically different from virgin PET (95.3 ± 2.4%, p>0.05), consistent with the biocompatibility standards established for PET copolymer cardiac-assist devices (see Ref. [17]). Catalytic-pyrolysis-recycled PET had a viability of 92.8 ± 2.8% and was Grade-0 responsive. Thermochemical-recycled PET had a viability of 87.4 ± 3.5% and was classified as Grade-1 (mild cytotoxicity) due to trace by-products from high-temperature pyrolysis.

All recycled samples produced haemolysis rates well below the 5% ISO 10993-4 threshold: enzymatic 1.82 ± 0.28%, catalytic 2.14 ± 0.35%, and thermochemical 3.26 ± 0.42%. Endotoxin levels remained below the medical-device limit of 0.5 EU/mL for all samples (0.10–0.18 EU/mL), and SEM images showed that erythrocytes on the surface retained their biconcave-disc shape without aggregation. Subcutaneous implantation in Sprague–Dawley rats for more than 4 weeks produced fibrous-capsule thicknesses of 85 ± 12 μm (enzymatic) and 98 ± 15 μm (catalytic-pyrolysis); neither was significantly different from virgin PET (78 ± 10 μm; p>0.05), indicating good in vivo tolerance for Class-II and selected Class-III device applications.

#### 3.5.2. Antibacterial Performance and Degradation

PET is not inherently antibacterial, but a rougher surface can prevent bacterial attachment. Enzymatic-recycled PET produced the smoothest surface (Ra 0.28 ± 0.04 μm) and showed the lowest bacterial-adhesion densities of $\left(2.3\pm 0.4\right)\times 10^{4}$ CFU/cm^2^ for *Staphylococcus aureus* and $\left(1.8\pm 0.3\right)\times 10^{4}$ CFU/cm^2^ for *Escherichia coli*, comparable to the antibacterial performance reported for chitosan-coated rPET electrospun scaffolds (Ref. [18]). Thermochemical-recycled PET had a relatively rougher surface (Ra 0.56 ± 0.08 μm) and thus permitted an approximately 40% increase in bacterial adhesion. Plasma surface modification or antibacterial coatings can be applied to further reduce colonisation of high-risk implants.

In vitro degradation in PBS (pH 7.4, 37 °C, 12 weeks) showed that all chemically recycled PET had good stability. Mass loss was below 1.4% per sample, and molecular-weight retention exceeded 96%. The buffer pH dropped by no more than 0.2 units, so no acidic degradation products were produced at concentrations high enough to cause peri-implant inflammation. SEM analysis after 12 weeks found only minor surface erosion and no damage to the overall structure. FTIR analysis showed that most degradation occurred in the amorphous region, while the crystalline part was unchanged, so the material still met the requirement for extended use under body-fluid stress. Together, these antibacterial-adhesion and degradation profiles show that chemically recycled PET is suitable for short- and medium-term medical implants, and the surface engineering established here can be extended to higher-acuity device classes.

## 4. Discussion

### 4.1. Comparison of Chemical Recovery Methods

The three chemical recovery technologies have different mechanisms, performance, and suitability for biomedicine. Alcoholysis with zinc acetate as the catalyst for catalytic pyrolysis obtained TPA with a recovery of 92.3% and a purity of 98.2% [24]. Zinc ions coordinate to the carbonyl carbon and reduce the activation energy from 125 kJ/mol to 68 kJ/mol through nucleophilic substitution. XPS analysis showed an unaltered chemical structure and an atomic ratio of C/O = 2.48 (theoretical 2.50). Zinc acetate outperforms zinc oxide because it is more soluble; in addition, due to its stronger Lewis-acid character and homogeneous speciation of Zn^2+^, it can coordinate with the ester carbonyl oxygen more effectively and lower the barrier for transesterification. As shown by the XPS Zn 2p binding-energy shift and the metal-salt glycolysis literature [22], this coordination-induced activation is also a factor. Biocompatible Mg-Fe layered double hydroxide catalysts similarly exploit Lewis-acid activation and achieved near-quantitative PET conversion at 200 °C [25], further corroborating the mechanistic importance of metal-ion coordination in glycolytic depolymerisation. End-group analysis showed a carboxyl content of 28.5 ± 2.1 μmol/g, the same as virgin PET, and only one recrystallisation step was required to achieve medical-grade purity at 94% efficiency. The robustness of the single-step recrystallisation arises from a large solubility difference between TPA and its main impurities in the hot-water/ethanol system: TPA crystallises selectively upon cooling, while more soluble mono-acid intermediates, oligomers, and residual Zn^2+^ (which remain as soluble acetate/glycolate complexes) are excluded from the crystal, and coloured degradation products are removed by an activated-carbon polish before crystallisation. Monomer purity consistently exceeded the 97% medical-grade level and reduced Zn^2+^ below the USP limit, as shown by the paired ICP–MS measurements in Section 2.6.

Thermochemical recovery is a free-radical chain reaction that generates a complex mixture of products, the proportions of which change significantly with temperature; bis(2-hydroxyethyl) terephthalate forms at 450 °C, while terephthalic acid is relatively abundant at 550 °C. This route alters the material’s properties more substantially than alcoholysis: crystallinity is reduced to 26.8% and oxygen permeability increases by 28%. Distillation, extraction, and other complex purification methods account for about 40% of the total processing costs, so unit production economics are low and the material is only suitable for non-biomedical uses.

Enzymatic recovery exploits the substrate specificity of PETase at low temperatures. Alkaline pretreatment increased enzyme adsorption by 3.8-fold through the expansion of the accessible surface area, and supplementary MHETase reduced product inhibition and increased the reaction rate by about 35% (see Refs. [9,10] for the fundamental enzyme characterisation underpinning these results). LC–MS/MS showed no organic impurities above 500 Da. Based on protein-adsorption studies, enzymatic-recycled PET adsorbed 0.42 ± 0.06 μg/cm^2^ of bovine serum albumin, approximately the same as virgin material. Circular-dichroism analysis showed that proteins maintained a near-native structure at the enzyme surface (58% α-helix) and the catalytic surface (55% α-helix), but were partially denatured at the thermochemical surface (42% α-helix). Based on these observations, enzymatic recovery is the most biocompatible route, whereas catalytic pyrolysis offers a trade-off between purity, throughput, and biological performance and is therefore chosen for mainstream applications.

### 4.2. Application in the Production of Biomedical Materials

Enzymatic- and catalytic-pyrolysis-recycled PET meet the requirements for Class-II and some Class-III devices, such as vascular prostheses, heart-valve suture rings, and hernia-repair patches [26,27]. Dynamic mechanical analysis of vascular prostheses showed that catalytic-pyrolysis-recycled PET has a storage modulus of 1.82 ± 0.08 GPa at physiological conditions (37 °C, 1 Hz), close to that of human arteries (1.5–2.0 GPa). Fatigue tests for a 10-year cardiac cycle showed less than an 8% reduction in performance. The density of platelet adhesion after 4 h of in vitro blood circulation was only $\left(4.8\pm 0.9\right)\times 10^{4}$ cells/cm^2^, indicating a low thrombogenic risk.

Electrospun nanofibre membranes (200–800 nm fibre diameter) loaded with hydrophilic amoxicillin at 12.3 ± 0.8 wt% and hydrophobic ibuprofen at 18.7 ± 1.2 wt% were prepared for drug-delivery systems [28]. Catalytic-pyrolysis-recycled PET retained more terminal functional groups and was thus chemically modified to extend drug release by PEG grafting to 60 days. For tissue engineering, 3D-printed porous scaffolds (200–500 μm pores, 65–75% porosity) supported human mesenchymal stem-cell densities of $\left(8.5\pm 0.7\right)\times 10^{5}$ cells/cm^3^ and maintained >95% viability after 7 days. RT-PCR showed no difference in osteogenic-marker gene expression from the control.

Several problems remain. Raw-material standardisation presents a first-hand challenge; medical waste is complex and has a 15–20% batch-to-batch variation. A protein concentration above 0.5% reduces the activity of the zinc-acetate catalyst by 35% and requires pretreatment. Regulatory approval entails an all-encompassing biological-evaluation cost of USD 500,000–USD 1 million over 2–3 years. To meet the additional requirement of implant-grade quality, a polishing train is needed after the single recrystallisation— activated-carbon decolourisation, sub-micron (0.22 μm) filtration, ion-exchange removal of residual Zn^2+^, and a depyrogenation step to keep endotoxins below 0.5 EU/mL—which together add about 12–18% to the unit production cost. A simplified process flow (waste collection → sterilisation → depolymerisation → activated-carbon/ion-exchange polishing → recrystallisation → depyrogenation → drying) and the corresponding cost breakdown support the techno-economic estimates. Based on economic analysis, at the current stage of technology, recycled PET costs 20–30% more than virgin PET and is not economically feasible unless crude-oil prices exceed USD 80/barrel or carbon taxes are introduced [29,30,31]. Market-acceptance surveys indicate that 42% of healthcare professionals and patients are fully willing to use recycled-material implants; therefore, enhancing the transparency of quality-control reports is needed to encourage widespread application.

## 5. Conclusions

This paper has built a complete technical route and a quality-control system for the chemical recycling of medical-grade PET, and shown that catalytic pyrolysis, thermochemical recovery, and enzymatic hydrolysis can all be applied to specific biomedical applications. Catalytic pyrolysis with zinc acetate showed the best overall technical performance (TPA recovery of 92.3 ± 1.8%, product purity of 98.2 ± 0.5%, ≥97% retention of virgin-PET tensile strength and elastic modulus) and is thus highly suitable for mainstream medical-grade applications. Enzymatic recovery took 24 h but demonstrated excellent biocompatibility (L929 viability 94.1 ± 2.2%, haemolysis 1.82 ± 0.28%, narrowest molecular-weight distribution) and a 46.5% reduction in carbon footprint; it was therefore chosen for the Class-III implantable-device route. Thermochemical recovery has a reaction time of 1 h and is catalyst-free, making it economically feasible for large-scale processing of low-value medical waste streams.

All three chemical paths produced recycled PET that met the ISO 10993 biocompatibility standards (cytotoxicity grade 0–1; endotoxin < 0.5 EU/mL) and had M_n_ values between 21,200 and 24,100 g·mol^−1^, significantly higher than those of mechanically recycled PET (18,600 ± 1420 g·mol^−1^). Techno-economic analysis showed a 2.6- to 3.8-year payback period and an IRR of 22–28%, demonstrating industrial feasibility (Refs. [19,20]). Future work will aim to reduce the production cost of enzymes from about USD 150/kg to less than USD 50/kg, develop bifunctional MOF-based catalysts that operate at lower temperatures, and build a comprehensive regulatory system for recycled medical-grade PET. With continued development of technology and supportive policies, by 2035 the realistic goal of more than 70% medical-grade PET recycling could be reached, simultaneously achieving environmental protection, economic benefits, and safeguarding of public health. 

## Figures and Tables

**Table 1:** Optimised conditions and recovery efficiency of three chemical recycling routes for medical-grade PET.

Route	Conditions	TPA (%)	Purity (%)	*t* (h)
Cat. (Zn(OAc)_2_)	200 °C, 3%, 6:1	92.3 ± 1.8	98.2 ± 0.5	4
Cat. (ZnO)	200 °C, 3%, 6:1	87.6 ± 2.3	97.1 ± 0.6	4
Cat. ([BMIM]Cl)	190 °C, 3%, 6:1	78.4 ± 3.1	95.4 ± 0.9	4
Thermochemical	500 °C, 10 °C/min	71.2 ± 3.8	92.4 ± 1.1	1
Enzymatic	35 °C, pH 8.0, 150 U/g	85.7 ± 2.7	97.8 ± 0.6	24

**Table 2:** Scale-up performance of the catalytic-pyrolysis recycling process at three reactor sizes.

Parameter	Lab. (100 mL)	Small (1 L)	Pilot (10 L)	Target
TPA recovery (%)	92.3 ± 1.8	90.5 ± 2.1	88.7 ± 2.4	>85
Product purity (%)	98.2 ± 0.5	97.6 ± 0.7	96.8 ± 0.9	>95
CV (%)	1.95	2.32	2.71	<5
Reaction time (h)	4.0	4.2	4.5	≤6
Throughput (g h^−1^)	5	50	500	–
Energy (kWh kg^−1^)	1.85	1.62	1.48	<2.0

**Table 3:** Molecular weight (GPC) and mechanical properties of virgin and recycled PET.

Sample	M_n_ (g·mol^−1^)	M_w_ (g·mol^−1^)	PDI	[η] (dL/g)	Tensile (MPa)	E (GPa)
Virgin PET	24,500 ± 520	48,200 ± 830	1.97 ± 0.04	0.82 ± 0.02	70.2 ± 1.9	2.48 ± 0.06
Catalytic-pyrolysis recycled PET	23,800 ± 610	47,500 ± 920	2.00 ± 0.05	0.80 ± 0.03	68.5 ± 2.3	2.42 ± 0.08
Thermochemical recycled PET	21,200 ± 980	44,300 ± 1250	2.09 ± 0.08	0.74 ± 0.04	62.3 ± 3.1	2.25 ± 0.11
Enzymatic-recycled PET	24,100 ± 480	47,800 ± 760	1.98 ± 0.03	0.81 ± 0.02	67.8 ± 2.1	2.45 ± 0.07
Mechanically recycled PET (ref.) ^1^	18,600 ± 1420	39,400 ± 2180	2.12 ± 0.11	0.65 ± 0.06	54.6 ± 3.8	2.08 ± 0.14

^1^ Reference values for mechanically recycled PET are taken from the literature for comparison [16].

**Table 4:** Biocompatibility test of recycled PET material.

Sample	L929 Viability (%)	Haemolysis Rate (%)	Endotoxin (EU/mL)	Cytotoxicity Grade	Capsule Thickness (μm)
Virgin PET	95.3 ± 2.4	1.65 ± 0.24	0.08 ± 0.02	0	78 ± 10
Catalytic-pyrolysis recycled PET	92.8 ± 2.8	2.14 ± 0.35	0.12 ± 0.03	0	98 ± 15
Thermochemical recycled PET	87.4 ± 3.5	3.26 ± 0.42	0.18 ± 0.04	1	125 ± 18
Enzymatic-recycled PET	94.1 ± 2.2	1.82 ± 0.28	0.10 ± 0.02	0	85 ± 12
ISO 10993 limit	≥70	<5	<0.5	0–1	–

## Data Availability

The data in this paper are available upon request from the corresponding author. The data are not publicly available at present due to ongoing research.
